# Plasma neutrophil gelatinase-associated lipocalin independently predicts dialysis need and mortality in critical COVID-19

**DOI:** 10.1038/s41598-024-57409-z

**Published:** 2024-03-20

**Authors:** Jonas Engström, Hazem Koozi, Ingrid Didriksson, Anders Larsson, Hans Friberg, Attila Frigyesi, Martin Spångfors

**Affiliations:** 1 Department of Clinical Sciences, Lund University, Anesthesiology and Intensive Care, Lund, 221 00 Sweden; 2 Department of Anesthesia and Intensive Care, Kristianstad Hospital, Kristianstad, 291 85 Sweden; 3 Department of Intensive and Perioperative Care, Skåne University Hospital, Malmö, 205 02 Sweden; 4 Department of Medical Sciences, Clinical Chemistry, Uppsala University, Uppsala, 751 05 Sweden; 5 Department of Intensive and Perioperative Care, Skåne University Hospital, Lund, 221 85 Sweden

**Keywords:** Predictive markers, Prognostic markers, Viral infection, Prognosis, Continuous renal replacement therapy, Haemodialysis, Predictive markers, Prognostic markers, Continuous renal replacement therapy, Haemodialysis, Acute kidney injury, Predictive markers, Prognostic markers, Viral infection, Prognosis, Continuous renal replacement therapy, Haemodialysis, Predictive markers, Prognostic markers, Continuous renal replacement therapy, Haemodialysis, Acute kidney injury

## Abstract

Neutrophil gelatinase-associated lipocalin (NGAL) is a novel kidney injury and inflammation biomarker. We investigated whether NGAL could be used to predict continuous renal replacement therapy (CRRT) and mortality in critical coronavirus disease 2019 (COVID-19). This prospective multicenter cohort study included adult COVID-19 patients in six intensive care units (ICUs) in Sweden between May 11, 2020 and May 10, 2021. Blood was sampled at admission, days two and seven in the ICU. The samples were batch analyzed for NGAL, creatinine, and cystatin c after the end of the study period. Initiation of CRRT and 90-day survival were used as dependent variables in regression models. Of 498 included patients, 494 were analyzed regarding CRRT and 399 were analyzed regarding survival. Seventy patients received CRRT and 154 patients did not survive past 90 days. NGAL, in combination with creatinine and cystatin c, predicted the subsequent initiation of CRRT with an area under the curve (AUC) of 0.95. For mortality, NGAL, in combination with age and sex, had an AUC of 0.83. In conclusion, NGAL is a valuable biomarker for predicting subsequent initiation of CRRT and 90-day mortality in critical COVID-19. NGAL should be considered when developing future clinical scoring systems.

## Introduction

The novel biomarker neutrophil gelatinase-associated lipocalin (NGAL) shows great promise as a prognostic indicator in COVID-19 patients in the emergency department^1,2^ and ICU^3–5^ as well as in other critically ill patients^6–11^. Being present in many tissues, NGAL is a marker for inflammation and kidney injury^12,13^. As opposed to the currently used and accepted markers of kidney function—such as creatinine and cystatin c—NGAL is not a marker of filtration but rather the injury itself. Therefore, NGAL is detectable before any elevation in creatinine occurs following kidney injury^14,15^, reporting tubular stress in real-time in animal models^16^. A similar quick response is seen in clinical studies where the time of renal insult is known, such as cardiopulmonary bypass during surgery^17–20^ and in conjunction with contrast administration during percutaneous coronary intervention^21^. A link between NGAL and the clinically important outcomes of renal replacement therapy (RRT) initiation and in-hospital death has also been show in cardiac surgery patients^22^, as well as the potiential to improve existing clinical scoring systems^23^.

In the outpatient setting, NGAL helps detect Kidney Disease Improving Global Outcomes (KDIGO) stage progression in chronic kidney disease^24^. In the emergency department, NGAL has been associated with subsequent RRT and 90-day mortality for patients with COVID-19^1,2^ and in-hospital mortality for patients without COVID-19^14^.

In critically ill patients, NGAL predicts acute kidney injury (AKI)^6–10^, and predicts mortality in patients with systemic inflammatory response syndrome (SIRS)^11^.

In patients undergoing RRT, NGAL predicts mortality^25,26^. As is the case in patients with cardio-renal syndrome or with AKI and underlying cirrhosis, where NGAL has also been suggested to be indicative of disease progression^27,28^.

NGAL is elevated in asymptomatic SARS-CoV-2 infection^21^. Furthermore, NGAL has been associated with histopathologic kidney injury, loss of kidney function, dialysis, shock, prolonged hospitalization, and in-hospital death in COVID-19 patients presenting to the emergency department^1^ as well as disease severity, critical illness, AKI, in-hospital mortality, 6-week mortality,^3,4,29,30^ and subsequent RRT in non-critical hospitalized COVID-19 patients^3,29^. Urinary NGAL has shown potential to predict AKI and mortality^31,32^ in critically ill COVID-19 patients. Length of mechanical ventilation has also been linked to NGAL^32^.

Plasma NGAL levels are comparable in patients dying without AKI to those diagnosed with AKI. It has therefore been hypothesized that NGAL measured in plasma mainly indicates general disease severity rather than kidney injury^33^. Plasma and urinary NGAL levels have been shown to have similar discriminative performance and sensitivity for AKI requiring dialysis^34^, although exhibiting different temporal profiles—with urinary NGAL performing better at predicting mortality or dialysis when measured as a peak value during the first 24 h following admission and the optimal temporal window for sampling both urinary and plasma NGAL being approximately 6–24 h after emergency department presentation^35^.

As blood is more frequently sampled during ICU care, determining whether these results also hold in plasma would be of clinical value, especially as the utility of plasma NGAL has previously been questioned as a marker of AKI in ICU patients due to it being of mainly neutrophil rather than renal origin^36^. Even though elevation of urinary NGAL in AKI is associated with a neutrophil component in animal models^37^, the opposite might not be true in plasma.

Therefore, we aimed to investigate the prognostic utility of plasma NGAL in critically ill COVID-19 patients. Our primary objective was to assess NGAL as a predictor of subsequent initiation of CRRT. As NGAL is an early marker associated with kidney injury, we hypothesized that it would improve prediction compared to conventional tests as part of a multivariable model. The secondary objective was to assess NGAL as a predictor of mortality.

## Methods

This prospective multicenter cohort study used data from the Swecrit-Covid-IR database, described more in-depth by Didriksson et al.^38^. The study is part of the more comprehensive Swecrit project, as summarized by Frigyesi et al.^39^.

Data was collected from patients admitted between May 11, 2020 and May 10, 2021. Except for two patients discharged to other geographical regions and lost to follow-up and one patient whose last follow-up occurred 9 days after intensive care unit (ICU) admission, all survivors were monitored for at least 101 days after ICU admission with a review of vital status. Additional blood samples were also collected at set time intervals, where possible.

Participants were recruited from ICUs in four hospitals in Skåne in southern Sweden, out of which two were university hospitals. Eligible for inclusion were adults admitted for laboratory-confirmed COVID-19 during the study period. The study was conducted in accordance with the Declaration of Helsinki, relevant guidelines, and regulations. Written informed consent was obtained from study participants at admission or at three or twelve-month follow-up. Due to the minimal patient risk presented by study participation, since no intervention was carried out, informed consent from non-survivors who had not been able to consent on admission, due to their illness, was waived. In these cases, the next of kin were informed and allowed to opt out for their late relatives. The study protocol was approved by the Swedish Ethical Review Authority (Dnr 2020-011955).

Blood samples were drawn from patients on ICU admission and on days two and seven in the ICU. Further blood samples were collected at three and twelve-month follow-up visits. Data on demographics, CRRT initiation, and survival until hospital discharge were collected during each patient’s ICU stay and later compiled from medical records. The 90-day survival of discharged patients was determined using the national Swedish population register.

Samples were collected in ethylenediaminetetraacetic acid (EDTA) treated test tubes, aliquoted following centrifugation and stored at -80 degrees Celsius. They were batch-analyzed for NGAL, creatinine, and cystatin c following the end of the data collection period. NGAL was analyzed using a commercial sandwich ELISA (DY1757, R &D Systems, Minneapolis, MN, USA) according to the manufacturer’s recommendations. The total coefficient of variation for the NGAL assay was approximately 6%. Creatinine and cystatin c were analyzed on a Mindray BS380 chemistry analyzer (Mindray Medical International, Shenzhen, China) using IDMS traceable enzymatic creatinine reagents from Abbott Laboratories (Abbott Park, IL, USA) and particle-enhanced turbidimetric cystatin c reagents from Gentian (Moss, Norway).

Since tests were analyzed after the study period had ended, treating clinicians were blinded to all NGAL results, but only partially to creatinine and cystatin c, as these are used in clinical practice. Laboratory staff analyzing tests were blinded to clinical state.

Cases with missing data were excluded from the analysis. Due to their skewness, the creatinine, NGAL, and cystatin c levels were log10 transformed. The difference in NGAL measured on day zero and day two of the ICU stay was calculated by subtracting the former from the latter. The calculated value is referred to as $\Delta _{\textrm{NGAL}}$ in this report.

Statistical analyses were performed using R^40^. For hypothesis tests, a level of significance of 5% was used. Several binomial linear candidate models were fitted with and without splines and interactions between the independent variables, using R’s built-in stats package. The area under the curve (AUC) for the receiver operating characteristic curves (ROC) and Brier scores were calculated for comparison, using the pROC^41^ and yardstick^42^ packages respectively. The areas under the ROC curves were compared using the method described by DeLong et al.^43^ using the pROC package. Models with a larger area under the ROC curve were considered superior when selecting candidate models. To further describe the difference between selected models, the integrated discrimination improvement (IDI)^44^ was calculated, comparing the best models including NGAL to the best models excluding NGAL.

In spline models, knots set to the first quartile, the median, and the third quartile were compared to algorithmically chosen knots with four degrees of freedom. Whichever gave the best apparent performance was used in the final model.

Since the study analyzed subsequent CRRT initiation and 90-day mortality, patients with missing data required for one analysis could be included in the other analysis, given that sufficient data were available.

To characterize the patient population, percentages were calculated for categorical variables; similarly, means and standard deviations (SD) were calculated for continuous variables. To compare the characteristics of patients receiving CRRT versus those not receiving CRRT and survivors versus non-survivors, the $\chi ^{2}$ test was used for categorical variables and the Welch test was used for continuous variables.

To illustrate a possible correlation between NGAL and creatinine in plasma in the study population, locally estimated scatterplot smoothing (LOESS) regression was calculated using the ggplot2 package^45^ after removing all values under the $1{\textrm{st}}$ and over the $99{\textrm{th}}$ percentile of either measurement to reduce the influence of outliers.

To illustrate a possible correlation between plasma NGAL and mortality, 90-day survival was recoded as 0 and 90-day non-survival as 1, after which LOESS regression was calculated for plasma NGAL vs survival. The difference in mean plasma NGAL concentration on admission of both groups was compared using a one-sided Student’s t-test.

To illustrate the performance of our mortality model, the population was stratified into tertiles based on predicted risk of death, after which a Kaplan-Meier plot for each tertile was plotted using the survival package^46^. The net reclassification improvement (NRI)^44^ for the same risk strata was calculated, comparing it to the best model not using NGAL.

## Results

**Figure 1:**
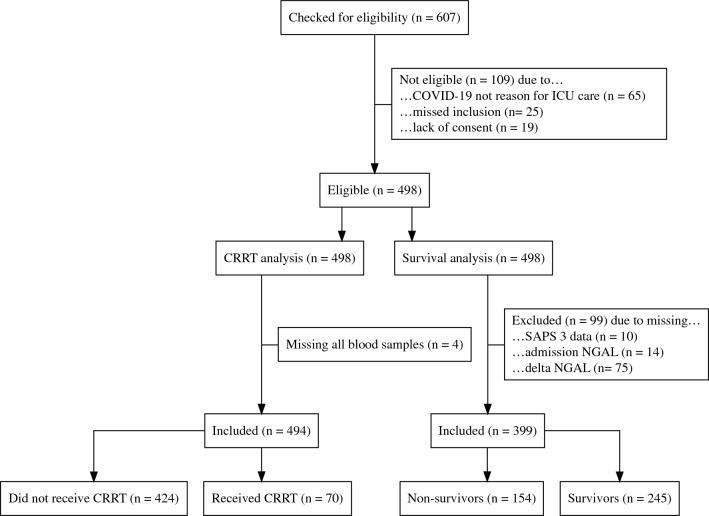
Flow chart. COVID-19, Coronavirus Disease 2019. CRRT, Continuous Renal Replacement Therapy. SAPS, Simplified Acute Physiology Score.

Of 607 patients screened, 65 were in the ICU for reasons other than their laboratory-confirmed COVID-19, 25 were missed for inclusion, and 19 did not consent. The 498 patients remaining were found eligible for inclusion; 494 patients could be analyzed for CRRT initiation, and 399 patients could be analyzed for survival after removing incomplete cases. Flow chart, see Fig. 1.

Of the patients missing NGAL data, 16 did not survive until the second sample could be obtained. An additional 36 patients had been discharged from the ICU before the second sample, possibly causing the second sample to be missed in the ward receiving the patient after ICU discharge. Three of the patients missing SAPS 3 data were admitted from ICUs at hospitals outside the study region, and the rest have no data registered on the exact route of admission to the ICU.

Of the 494 patients in the CRRT analysis, 70 received CRRT in the ICU. Of the 399 patients in the mortality analysis, 154 did not survive beyond 90 days. Patient characteristics are summarized in Tables 1 and 2.

**Table 1:** Characteristics of patients analyzed for initiation of continuous renal replacement therapy (CRRT).

	Overall	No CRRT	CRRT	p
General characteristics				
n	494	424	70	
Age (years)	63 (13)	63 (13)	64 (11)	0.545
Male (%)	74	73	80	0.251
Body mass index	31 (7)	31 (6)	33 (7)	**0.013**
Clinical frailty scale	3 (1)	3 (1)	3 (1)	0.519
Charlson comorbidity index	3 (2)	3 (2)	3 (2)	0.142
Hypertension (%)	55	52	71	**0.005**
Complicated diabetes (%)	14	11	31	< **0.001**
Cardiovascular disease (%)	23	22	30	0.199
COPD (%)	19	20	11	0.123
Liver disease (%)	1	1	1	1.000
CKD (%)	4	2	14	< **0.001**
Status and laboratory values on ICU admission				
SAPS 3	60 (14)	60 (14)	63 (13)	0.082
SOFA	7.4 (3.3)	7.3 (3.2)	8.2 (3.7)	**0.042**
Respiratory SOFA	3.7 (0.6)	3.6 (0.6)	3.8 (0.4)	**0.031**
Cardiovascular SOFA	1.2 (1.6)	1.1 (1.5)	1.6 (1.7)	**0.028**
Neurologic SOFA	2.4 (1.9)	2.5 (1.9)	2.0 (1.9)	0.106
Coagulation SOFA	0.5 (1.0)	0.4 (0.9)	0.9 (1.4)	< **0.001**
Hepatic SOFA	0.1 (0.3)	0.1 (0.3)	0.1 (0.4)	0.446
Arterial pH	7.4 (0.1)	7.4 (0.1)	7.4 (0.1)	0.527
PaO_2_ (kPa)	9 (3)	9 (4)	9 (3)	0.488
P/F ratio (kPa/%)	22 (15)	22 (16)	19 (10)	0.219
NGAL (ng/ml)	160 (158)	148 (144)	243 (215)	< **0.001**
$\Delta _{\textrm{NGAL}}$ (ng/ml)	148 (1172)	154 (1053)	111 (1736)	0.792
Creatinine (${\upmu }$mol/l)	102 (105)	88 (50)	191 (240)	< **0.001**
Cystatin c (mg/l)	2 (1)	2 (1)	3 (2)	< **0.001**
Platelet count ($\times 10^{9}$)	280 (128)	289 (129)	233 (113)	**0.001**
Leukocyte count ($\times 10^{9}$)	12 (10)	12 (7)	14 (16)	**0.043**
Lymphocyte count ($\times 10^{9}$)	1 (4)	1 (1)	3 (10)	**0.002**
CRP (mg/l)	155 (85)	156 (85)	149 (84)	0.510
IL-6 (ng/l)	585 (3016)	618 (3270)	416 (902)	0.648
Ferritin (mg/l)	2231 (5343)	2234 (5660)	2212 (3122)	0.976
Bilirubin (${\upmu }$mol/l)	10 (6)	10 (6)	10 (8)	0.516
Therapies during ICU stay				
Invasive Ventilation (%)	72	67	100	< **0.001**
Tracheostomy (%)	16	13	34	< **0.001**
ECMO (%)	6	6	6	1.000
Complications				
Cardiac Arrest (%)	3	3	9	**0.029**
Outcomes				
Length of ICU stay (days)	14 (16)	12 (14)	26 (20)	< **0.001**
Length of hospital stay (days)	33 (33)	31 (30)	49 (40)	< **0.001**
ICU mortality (%)	30	26	57	< **0.001**
90-Day Mortality (%)	39	35	61	< **0.001**

Significant values are in [bold].

BPM, beats per minute; COPD, chronic obstructive pulmonary disease; CKD, chronic kidney disease; CRP, C reactive protein; ECMO, extracorporeal membrane oxygenation; ICU, intensive care unit; IL-6, Interleukin 6; NGAL, neutrophil gelatinase-associated lipocalin; PaO_2_, arterial partial pressure of oxygen; SAPS 3, simplified acute physiology score 3; SOFA, sequential organ failure assessment. Values are presented as means with standard deviations within parentheses unless otherwise specified.

**Table 2:** Characteristics of patients analyzed for survival.

	Overall	Survivors	Non-survivors	p
General characteristics				
n	399	245	154	
Age (years)	63 (13)	59 (13)	71 (9)	<**0.001**
Male (%)	74	74	75	1.000
Body mass index	31 (7)	32 (7)	30 (6)	**0.027**
Clinical frailty scale	2.9 (1.0)	2.8 (1.0)	3.1 (1.1)	**0.001**
Charlson comorbidity index	3 (2)	2 (2)	4 (2)	<**0.001**
Hypertension (%)	55	51	62	**0.040**
Complicated diabetes (%)	13	11	16	0.220
Cardiovascular disease (%)	22	14	34	<**0.001**
COPD (%)	20	19	23	0.408
Liver disease (%)	2	1	3	0.159
CKD (%)	3	3	3	1.000
Status and laboratory values on ICU admission				
SAPS 3	60 (13)	57 (13)	65 (12)	<**0.001**
SOFA	7.5 (3.2)	7.6 (3.1)	7.5 (3.3)	0.820
Respiratory SOFA	3.7 (0.5)	3.7 (0.5)	3.7 (0.4)	0.448
Cardiovascular SOFA	1.2 (1.5)	1.1 (1.5)	1.3 (1.6)	0.204
Neurologic SOFA	2.5 (1.9)	2.6 (1.9)	2.4 (1.9)	0.245
Coagulation SOFA	0.4 (1.0)	0.5 (1.1)	0.4 (0.9)	0.298
Hepatic SOFA	0.1 (0.3)	0.1 (0.3)	0.1 (0.3)	0.987
Arterial pH	7.4 (0.1)	7.4 (0.1)	7.4 (0.1)	0.411
PaO_2_ (kPa)	9 (3)	9 (3)	9 (2)	0.903
P/F ratio (kPa/%)	21 (13)	21 (10)	20 (17)	0.807
NGAL (ng/ml)	158 (142)	141 (117)	184 (172)	**0.003**
$\Delta _{\textrm{NGAL}}$ (ng/ml)	156 (1211)	70 (908)	294 (1573)	0.072
Creatinine (${\upmu }$mol/l)	102 (111)	101 (122)	103 (92)	0.854
Cystatin c (mg/l)	1.9 (1.2)	1.8 (1.2)	2.1 (1.1)	**0.002**
Platelet count ($\times 10^{9}$)	277 (123)	284 (123)	266 (123)	0.195
Leukocyte count ($\times 10^{9}$)	12 (9)	11 (6)	14 (12)	**0.003**
Lymphocyte count ($\times 10^{9}$)	1 (5)	1 (2)	2 (7)	0.221
CRP (mg/l)	158 (85)	158 (85)	156 (86)	0.835
IL-6 (ng/l)	611 (3235)	246 (1042)	1194 (5005)	**0.015**
Ferritin (mg/l)	2272 (5744)	2366 (6970)	2128 (3062)	0.705
Bilirubin (${\upmu }$mol/l)	10 (6)	10 (5)	11 (7)	0.080
Therapies during ICU stay				
CRRT (%)	15	9	25	<**0.001**
Invasive ventilation (%)	77	71	88	<**0.001**
Tracheostomy (%)	18	17	19	0.689
ECMO (%)	7	6	7	0.846
Complications				
Cardiac arrest (%)	3	1	6	**0.020**
Outcomes				
Length of ICU stay (days)	16 (16)	16 (18)	17 (13)	0.391
Length of hospital stay (days)	35 (34)	42 (41)	26 (17)	<**0.001**
ICU mortality (%)	30	0	78	<**0.001**

Significant values are in [bold].

BPM, beats per minute; COPD, chronic obstructive pulmonary disease; CKD, chronic kidney disease; CRP, C reactive protein; CRRT, continuous renal replacement therapy; ECMO, extracorporeal membrane oxygenation; ICU, intensive care unit; IL-6, interleukin 6; NGAL, neutrophil gelatinase-associated lipocalin; PaO_2_, arterial partial pressure of oxygen; SAPS 3, simplified acute physiology score 3; SOFA, sequential organ failure assessment. Values are presented as means with standard deviations within parentheses unless otherwise specified.

In our analysis of subsequent CRRT initiation, the group receiving CRRT had a a higher prevalence of preexisting hypertension (71% vs. 52%), complicated diabetes (31% vs. 11%), and chronic kidney disease (14% vs. 2%). On admission, the CRRT recipients had a higher mean plasma NGAL level (243 ng/ml, SD 215 ng/ml vs. 148 ng/ml, SD 144 ng/ml). Their mortality was higher both in the ICU (57% vs. 26%) and over 90 days (61% vs. 35%). (See Table 1).

In our analysis of mortality, the non-survivors had a higher mean clinical frailty scale value (3.1, SD 1.1 vs. 2.8, SD 1.0) and Charlson comorbidity index (4, SD 2 vs. 2, SD 2). On admission, the non-survivors had a higher mean plasma NGAL level (184 ng/ml, SD 172 ng/ml vs. 141 ng/ml, SD 117 ng/ml). They received CRRT to a larger degree than the survivors (25% vs. 9%). (See Table 2.)

**Figure 2:**
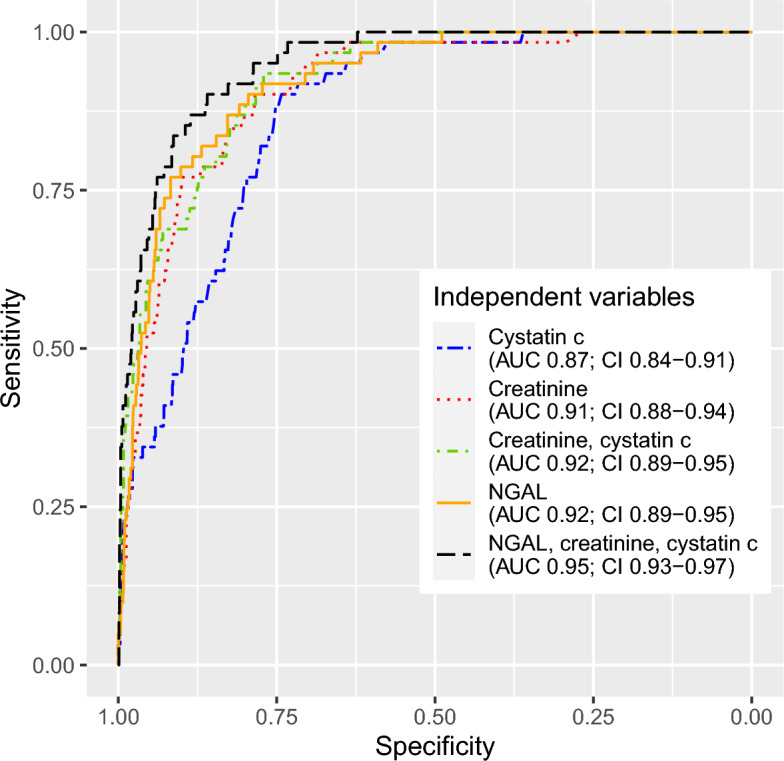
Receiver operating characteristics curves for predicting subsequent continuous renal replacement therapy. The areas under the curves were compared using one-sided DeLong’s test, yielding the following p-values: cystatin c vs. creatinine < 0.001, creatinine and cystatin c < 0.001, NGAL 0.002, NGAL, creatinine, and cystatin c < 0.001; creatinine vs. creatinine and cystatin c 0.021, NGAL 0.271, NGAL, creatinine, and cystatin c 0.001; creatinine and cystatin c vs. NGAL 0.500, NGAL, creatinine, and cystatin c 0.004; NGAL vs. NGAL, creatinine, and cystatin c 0.007. AUC, area under curve. CI, 95% confidence interval. NGAL, neutrophil gelatinase-associated lipocalin.

Our models for predicting subsequent CRRT initiation yielded AUCs ranging from 0.87 to 0.95. The model achieving the largest area under the ROC curve used NGAL combined with creatinine and cystatin c as independent variables (AUC 0.95). The IDI of the model, compared to the best model excluding NGAL—which used creatinine and cystatin c—was 7.4%. Figure 2 reports areas under the ROC curves. The differences in the areas under the curves were statistically significant, apart from NGAL alone (AUC 0.92) versus creatinine alone (AUC 0.91, p 0.271) and NGAL alone versus creatinine combined with cystatin c (AUC 0.92, p 0.500). Figure 2 reports areas under the ROC curves, including their confidence intervals. All p-values are reported in the caption of Fig. 2.

**Figure 3:**
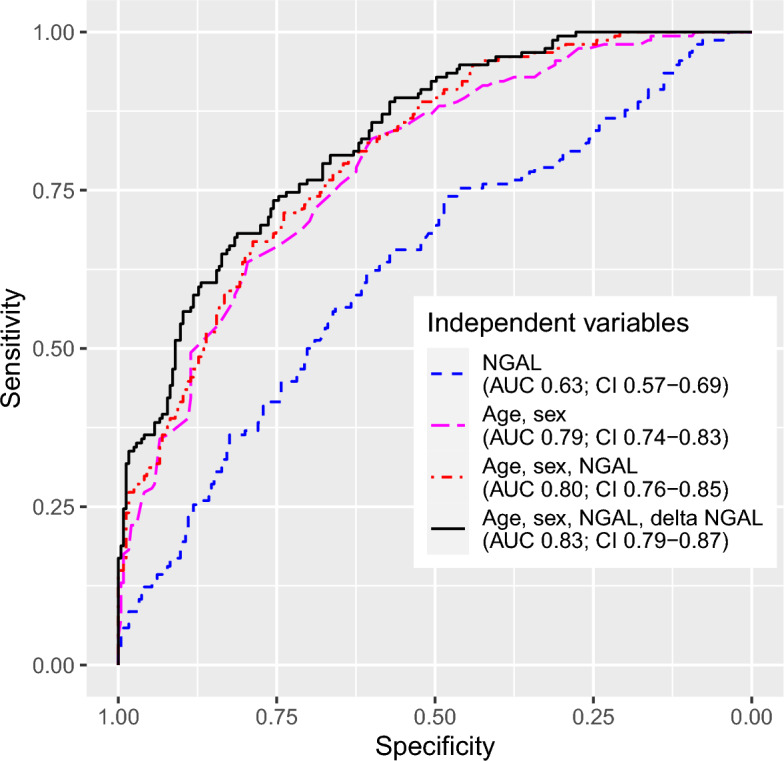
Receiver operating characteristics curves for predicting 90-day mortality. The areas under the curves were compared using one-sided DeLong’s test, yielding the following p-values: NGAL vs. age and sex < 0.001, age, sex, and NGAL < 0.001, age, sex, NGAL, and $\Delta _{\textrm{NGAL}}$ < 0.001; age and sex vs. age, sex, and NGAL 0.030, age, sex, NGAL, and $\Delta _{\textrm{NGAL}}$ 0.001; age, sex, and NGAL vs. age, sex, NGAL, and $\Delta _{\textrm{NGAL}}$ 0.005. AUC, area under curve. CI, 95% confidence interval. NGAL, neutrophil gelatinase-associated lipocalin.

Our models for predicting 90-day mortality yielded AUCs ranging from 0.63 to 0.83. The model achieving the largest area under the ROC curve used age, sex, NGAL on admission, and $\Delta _{\textrm{NGAL}}$ between day zero and two in the ICU as independent variables (AUC 0.83). The IDI of this model, when compared to the model using age and sex—which was the best model excluding NGAL—was 2.0%. When predicted risk was split into tertiles, the NRI calculated between the same two models was 8.1%. The next best AUC was the the model using age, sex, and NGAL as independent variables (AUC 0.80, p 0.005). Figure 3 reports areas under the ROC curves including their confidence intervals. All curves differed significantly. All p-values are reported in the caption of Fig. 3.

**Figure 4:**
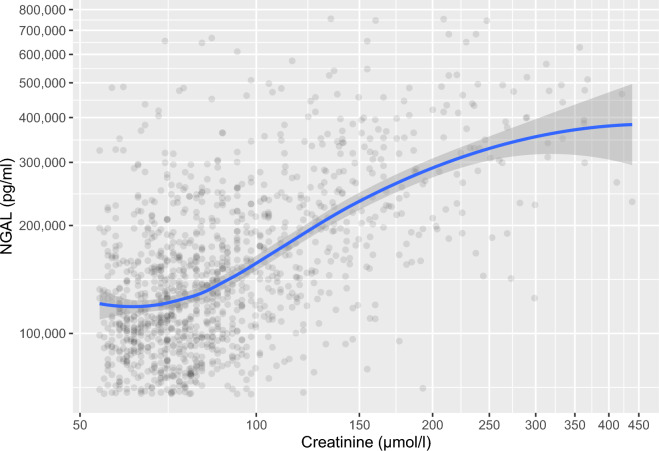
Correlation between neutrophil gelatinase-associated lipocalin (NGAL) and creatinine concentrations between the $1{\textrm{st}}$ and $99{\textrm{th}}$ percentiles. Blue line fitted to data using locally estimated scatterplot smoothing (LOESS) with the gray band showing the 95% confidence interval.

**Figure 5:**
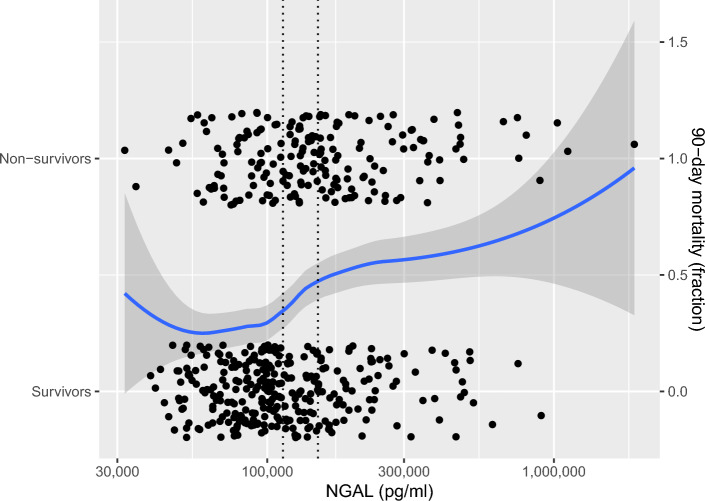
Admission neutrophil gelatinase-associated lipocalin (NGAL) vs. 90-day mortality. The dotted line represents the means of respective groups (difference in means p < 0.001, calculated using one-sided Student’s t-test). Blue line fitted to data using locally estimated scatterplot smoothing (LOESS) with the gray band showing the 95% confidence interval.

The relationships between NGAL and creatinine and 90-day mortality are plotted in Figs. 4 and 5. Regression lines in the figures were computed using LOESS. In Fig. 5, means were compared using a one-sided Student’s t-test.

**Figure 6:**
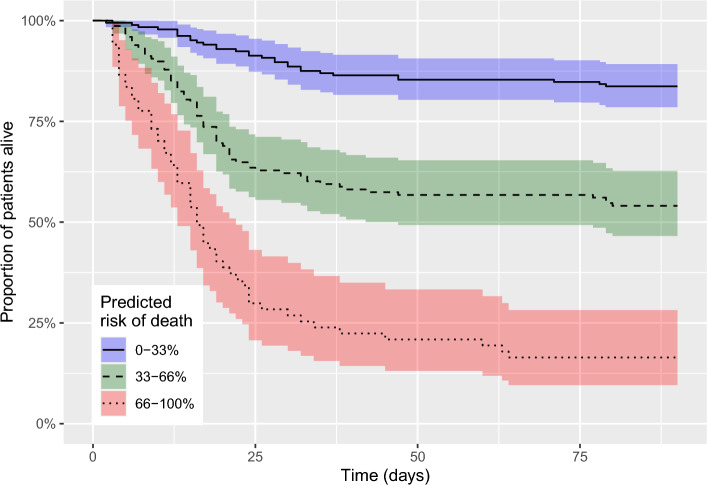
Kaplan-Meier plot showing actual 90-day survival stratified by predicted risk of death calculated using the best performing model in our study. The model uses age, sex, neutrophil gelatinase-associated lipocalin (NGAL), and $\Delta _{\textrm{NGAL}}$ between day zero and two in the intensive care unit. Bands show 95% confidence intervals. Table 3 shows the net reclassification improvement for the model, compared to the best model excluding NGAL, using the same risk strata.

Figure 6 represents Kaplan-Meier curves showing 90-day survival of patients stratified into tertiles of predicted mortality risk. The bands in the figure show 95% confidence intervals. Table 3 shows reclassification for the model, compared to the best model excluding NGAL, using the same risk strata.

**Table 3:** Predicted risks and reclassification of 90-day mortality risk using the best performing model in our study.

	Number of patients			Reclassified		Net correctly reclassified (%)
	0-33%	33-66%	66-100%	Increased risk (n)	Decreased risk (n)	
Predicted 90 day mortality risk with NGAL						
Patients with 90 day mortality (n = 154)						
Predicted 90 day mortality without NGAL						
0–33%	19	6	3	32	24	5.2
33–66%	10	49	24			
66–100%	1	13	29			
Patients without 90 day mortality (n = 254)						
Predicted 90 day mortality without NGAL						
0–33%	137	12	0	18	25	2.9
33–66%	17	60	6			
66–100%	0	8	5			
Net reclassification improvement						8.1

The model uses age, sex, neutrophil gelatinase-associated lipocalin (NGAL), and $\Delta _{\textrm{NGAL}}$ between day zero and two in the intensive care unit, compared to the best-performing model excluding NGAL, which uses age and sex.

## Discussion

Our study shows that plasma NGAL, in addition to conventional tests for kidney function, can improve the prediction of subsequent initiation of CRRT compared to conventional biomarkers alone in critical COVID-19. Furthermore, NGAL on ICU admission and an early increase in NGAL can improve mortality prediction compared to age and sex alone.

This is an important finding, as the rapid rise of NGAL in response to renal stress enables clinicians to start treatment promptly and avoid unnecessary deterioration—something essential in the critically ill whose physiologic reserves are already strained^47^.

When compared to models not using NGAL, the discriminatory ability for subsequent CRRT initiation of our model utilizing NGAL, creatinine, and cystatin c, as measured by the area under the ROC curve, increased less in relative terms than the discriminatory ability of our model utilizing age, sex, NGAL, and $\Delta _{\textrm{NGAL}}$ for prediction of mortality. In absolute terms, our CRRT model did, however, perform better than our mortality model, which implies that NGAL might be more useful for CRRT prediction in clinical practice.

Previous research has shown that NGAL in urine predicts mortality^31^ and subsequent initiation of CRRT^32^ in critical COVID-19. In the initial resuscitative phase of intensive care, patients do not always produce urine, making reliance on urine tests for rapid diagnostics unreliable. The finding that plasma NGAL can be used instead may also simplify testing, as blood samples are more routinely handled in the ICU, and NGAL can potentially be analyzed in the same samples as other tests. This increases its utility.

Our findings align with previous research in the area, showing the potential of NGAL as a diagnostic tool. Elevation in NGAL has been documented even in asymptomatic SARS-CoV-2 infection^21^. Despite this, NGAL has been found useful in assessing risk for dialysis^2^ and poor outcome^1^ in patients presenting to the emergency department with COVID-19. Severe inflammation does not seem to diminish its discriminatory capability for AKI^10^, although it may make tailoring of cut-off values and models necessary^48^.

A strength of our study was that we could follow up a large proportion of the surviving patients for more than three months, which enabled us to use 90-day mortality rather than less patient-centered, shorter-term survival as an outcome. The multicenter design included university and community hospitals which increases the generalizability of our results. The fact that we acquired sequential blood tests also enabled the analysis of dynamics in NGAL. A weakness was, however, the frequency of sampling. Our study sampled NGAL on days zero, two, seven, and 90, yielding a limited temporal resolution. Elevation of NGAL is detectable up to 48 hours before AKI diagnosis^8^ and dynamics over 48 hours have been linked to mortality^48^, it is possible that more frequent testing would have provided data for even better prediction models. Furthermore, our zero-day sample is from the day of ICU admission, i.e. when the patients had already developed critical illness. NGAL sampled before ICU admission should be considered for future studies, as this would also allow for the prediction of ICU need altogether.

Moreover, the analysis methods used in our study did not allow us to distinguish between monomeric NGAL, of renal origin, and dimeric NGAL, of neutrophil origin, which has previously been proposed as a way of improving diagnostics when measuring NGAL in urine^49^. Measurement of the different forms of NGAL and their ratio in blood might be valuable, and it is an area that warrants further investigation.

NGAL varies significantly with age and sex, with higher levels seen in females and older individuals^24^. Even though our mortality model considered this, as age is a strong predictor of mortality in COVID-19^38^, our CRRT model did not. Stratifying patients in different age and sex groups or looking at relative change or rate of change in NGAL rather than absolute levels could result in a superior model. However, the uneven male-to-female distribution in our study population did not lend itself to any far-reaching conclusions about the topic. A weakness of our study is also the fact that creatinine is used both to define AKI and as a guide when deciding to start CRRT, which can introduce biases.

The fact that NGAL did not significantly outperform creatinine individually but substantially improved diagnostics as part of a multivariable model implies that constructing such models may be more fruitful than finding a perfect individual prognostic marker. Even though NGAL is a good predictor of AKI, so are several other markers^6,7^. In an era where statistical models with the help of machine learning and artificial intelligence are ever more capable of complex pattern recognition, looking at interactions between several factors promises to become more and more relevant in the intensive care unit of tomorrow, e.g. combining NGAL with radiological data^4^.

It is also possible that improved diagnostics and more sensitive tests will require us to revisit the definition of conditions such as AKI^15^ or open up new therapeutic pathways to target^13^. However, as our study was mainly exploratory, further development and validation of clinical prediction models are needed.

In summary, our study shows that plasma NGAL predicts subsequent initiation of CRRT and mortality in critical COVID-19. Plasma NGAL may be valuable for future clinical scoring systems and prediction models.

## Data Availability

The datasets analyzed during the current study are available from the corresponding author upon reasonable request.
